# Protective Effects of *Chlorella*-Derived Peptide Against UVC-Induced Cytotoxicity through Inhibition of Caspase-3 Activity and Reduction of the Expression of Phosphorylated FADD and Cleaved PARP-1 in Skin Fibroblasts

**DOI:** 10.3390/molecules17089116

**Published:** 2012-08-02

**Authors:** Mei Fen Shih, Jong Yuh Cherng

**Affiliations:** 1 Department of Pharmacy, Chia-Nan University of Pharmacy and Science, Tainan 717, Taiwan; 2 Department of Chemistry and Biochemistry, National Chung Cheng University, Chia-Yi 621, Taiwan

**Keywords:** UVC, caspase-3, skin fibroblast

## Abstract

UVC irradiation induces oxidative stress and leads to cell death through an apoptotic pathway. This apoptosis is caused by activation of caspase-3 and formation of poly(ADP-ribose) polymerase-1 (PARP-1). In this study, the underlying mechanisms of *Chlorella* derived peptide (CDP) activity against UVC-induced cytotoxicity were investigated. Human skin fibroblasts were treated with CDP, vitamin C, or vitamin E after UVC irradiation for a total energy of 15 J/cm^2^. After the UVC exposure, cell proliferation and caspase-3 activity were measured at 12, 24, 48, and 72 h later. Expression of phosphorylated FADD and cleaved PARP-1 were measured 16 h later. DNA damage (expressed as pyrimidine (6-4) pyrimidone photoproducts DNA concentration) and fragmentation assay were performed 24 h after the UVC exposure. Results showed that UVC irradiation induced cytotoxicity in all groups except those treated with CDP. The caspase-3 activity in CDP-treated cells was inhibited from 12 h onward. Expression of phosphorylated FADD and cleaved PARP-1 were also reduced in CDP-treated cells. Moreover, UVC-induced DNA damage and fragmentation were also prevented by the CDP treatment. This study shows that treatment of CDP provides protective effects against UVC-induced cytotoxicity through the inhibition of caspase-3 activity and the reduction of phosphorylated FADD and cleaved PARP-1 expression.

## 1. Introduction

Oxidative DNA damage has been implicated in several human diseases, especially cancers [1,2] and neurodegenerative disorders [3]. Reactive oxygen species (ROS) generated either by environmental factors or as a consequence of normal metabolic processes, may damage DNA. Each living cell of an organism must repair thousands of ROS-induced lesions each day [4]. UVC (100~280 nm) is known to produce superoxide radicals, resulting in hydrogen peroxide generation. The hydrogen peroxide-derived hydroxyl radical reacts with cellular proteins and membrane lipids that provoke various physiological cellular responses or lead to apoptosis [5,6]. Normally, the skin responds to UV exposure with cell cycle arrest to allow for the repair of damaged cells or apoptosis if repair is not possible. UVC irradiation-induced apoptosis has been demonstrated through the activation of death receptor CD95 (Fas/APO-1) [7]. Upon activation, an adapter molecule, Fas associated death domain (FADD), is recruited and responsible for downstream signal transduction by recruitment of caspase-8, one of the key enzymes in the apoptotic pathway [8]. This subsequently activates caspase-3, one of the executive enzymes of apoptosis, and proceeds to internucleosomal fragmentation of DNA [9,10,11]. Another characteristic event of apoptosis is the proteolytic cleavage of poly(ADP-ribose)polymerase-1 (PARP-1), a nuclear enzyme involved in DNA repair, DNA stability, and transcriptional regulation. Cleaved PARP-1 facilitates cellular disassembly and serves as a marker of cells undergoing apoptosis [12,13]. Fibroblasts are responsible for synthesis and maintenance of the extracellular framework that provides structure support for various tissues and organs. The activity of fibroblast is particularly important during growth, and during healing following injury [14,15]. UVC induced apoptosis in fibroblasts has been demonstrated to be mediated through caspase-3 activation and cleavage of PARP-1 [16].

*Chlorella* has been shown to possess many biological effects, such as promoting the growth rate of animals [17], modulating immune function [18,19], preventing stress-induced ulcers [20], preventing high fat-diet induced dyslipidemia [21,22], and ameliorating streptozocin-induced hyperglycemic status of diabetes [23,24]. In addition, *Chlorella*-derived peptide (CDP) has been shown to inhibit UVB-induced MMP-1 levels in fibroblasts [25,26], and UVC-induced cytotoxicity [27]. However, the protective mechanisms of *Chlorella* against UVC-induced cytotoxicity have not been investigated. In this study, CDP activity on the UVC-induced apoptotic pathway, *i.e.*, phosphorylated FADD, caspase-3 activity, and cleaved PARP-1 were investigated.

## 2. Results and Discussion

### 2.1. Protective Effects of CDP Against UVC-Induced Cytotoxicity in Human Skin Fibroblasts

**Figure 1:**
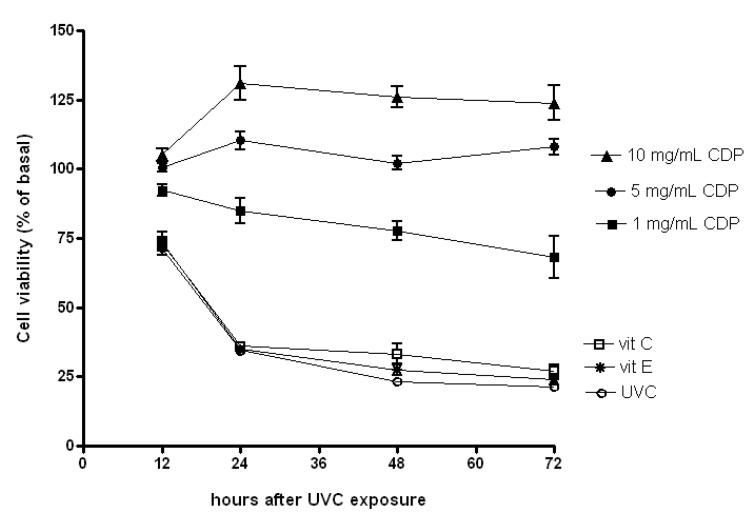
Protective effects of CDP on UVC-induced cytotoxicity. Skin fibroblasts were treated with CDP (1, 5 or 10 mg/mL), vitamin C (150 μM), or vitamin E (25μM) after UVC exposure with a total dose of 15 J/cm^2^ at 12, 24, 48, and 72 h prior to cell viability assay.

Exposure to UVC is known to induce cell apoptosis [7,28]. Our data showed that the fibroblasts retained about 75% cell viability compared to non UV-treated cells (referred to as basal) 12 h after UV exposure for UVC, vitamin E and C treated groups. Further the UVC irradiation induced more than 70% cell death in skin fibroblasts 24 h after the exposure (Figure 1). A large decrease in cell viability was also observed in those treated with vitamin E or C after the UV irradiation. However, fibroblasts treated with 1 mg/mL of CDP remained 90% in cell viability 12 h post the UV irradiation and only slightly reduced further at 24, 48 and 72 h. Interestingly, a treatment with CDP (5 and 10 mg/mL) showed 100% protection against UVC-induced cytotoxicity throughout the entire experimental period.

### 2.2. Inhibitory Effects of CDP on UVC-Induced Apoptosis Measured as Caspase-3 Activity, Phosphorylated FADD, and Cleaved PARP-1 Expression

**Figure 2:**
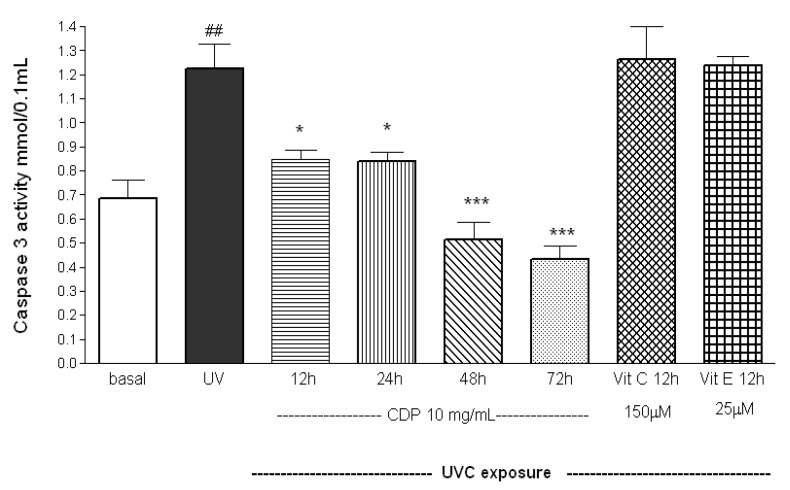
Inhibitory effects of CDP on UVC-induced caspase-3 activity. Skin fibroblasts were treated with CDP (10 mg/mL), vitamin C (150 μM), or vitamin E (25 μM) after UVC exposure with a total dose of 15 J/cm^2^ at 12, 24, 48, and 72 h prior to caspase-3 activity assay. Statistics are shown for UVC treated group, ^##^ *p* < 0.01 compared to basal group, ^*^ *p* < 0.05 compared to UVC group at 12 and 24 h after the UV exposure, ^***^ *p* < 0.005 compared to UVC group 48 and 72 h after the UV exposure.

Caspase-3 activity was determined at 12 h after the exposure in order to obtain enough cells for the non CDP-treated groups after UVC exposure. Caspase-3 activities were higher in the UV irradiated group than the basal 12 h after the UVC exposure (*p* < 0.01, Figure 2).

Antioxidants (vitamin E and C) had no preventive effect on UVC-induced caspase-3 activity. However, caspase-3 activities were significantly lower in CDP-treated (10 mg/mL) cells compared to the UV group at 12 and 24 h after the UV exposure (*p* < 0.05). The activities remarkably decreased when the fibroblasts were cultured with CDP for 48, and 72 h (*p* < 0.01).

**Figure 3:**
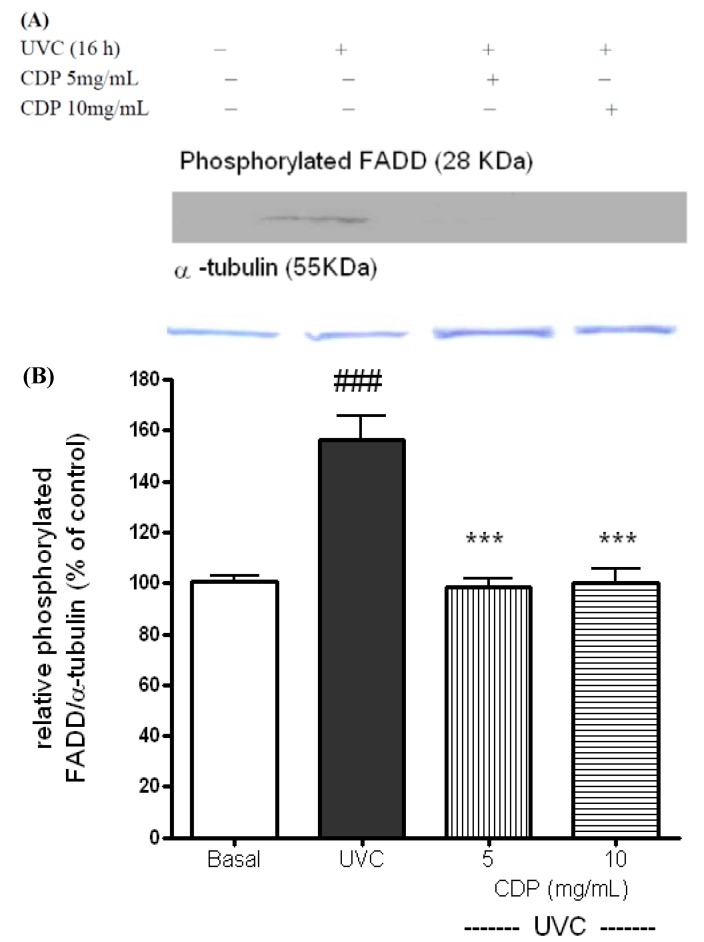
Effects of CDP on UVC-induced phosphorylated FADD protein expression (**A**). Skin fibroblasts were treated with CDP 5 and 10 mg/mL after UVC exposure with a total dose of 15 J/cm^2^ and cells were cultured at 16 h prior to harvesting for protein extraction. Statistics of relative density (**B**) are shown for UVC treated group, ^###^ *p* < 0.005 compared to basal group, ^***^ *p* < 0.005 compared to the CDP 5mg/mL and 10 mg/mL-treated groups.

UVC induced apoptosis has been shown via CD95 receptors [7] and subsequently activation of FADD [8]. The activation of FADD is in a phosphorylated form with molecular weight approximately 28 KDa. The phosphorylated FADD was shown distinctly expressed at 16 h after the UVC exposure (Figure 3), however the expression in CDP-treated groups (5 and 10 mg/mL) were suppressed than those in the UVC control.

**Figure 4:**
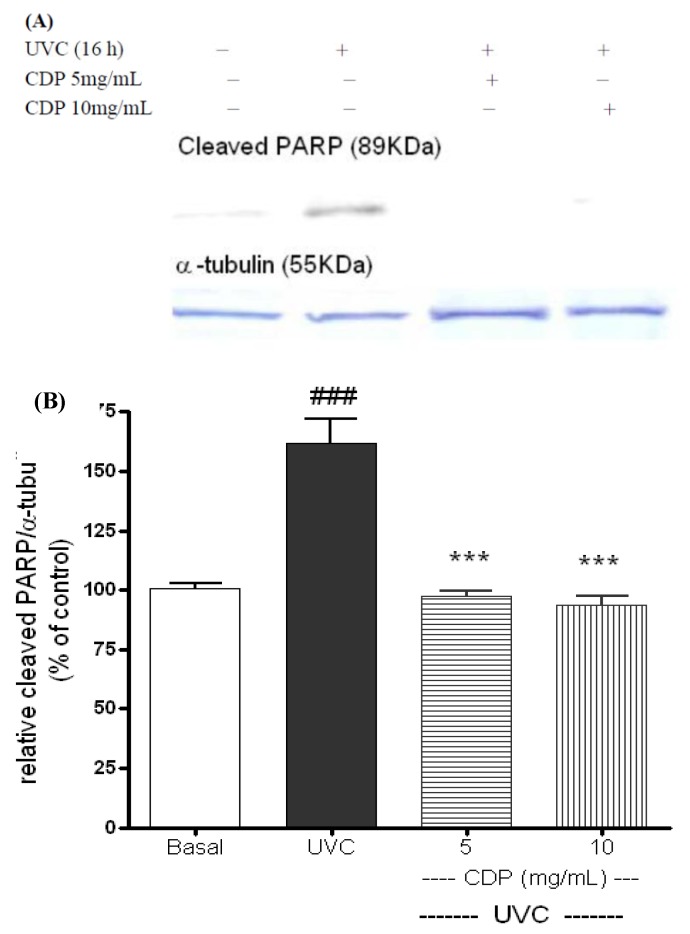
Inhibitory effects of CDP on UVC-induced cleaved PARP-1 protein expression (**A**). Skin fibroblasts were treated with CDP 5 and 10 mg/mL after UVC exposure with a total dose of 15 J/cm^2^ and cells were cultured at 16 h prior to harvesting for protein extraction. Statistics of relative density (**B**) are shown for UVC treated group, ^###^ *p* < 0.005 compared to basal group, ^***^ *p* < 0.005 compared to the CDP 5mg/mL and 10 mg/mL-treated groups.

**Figure 5:**
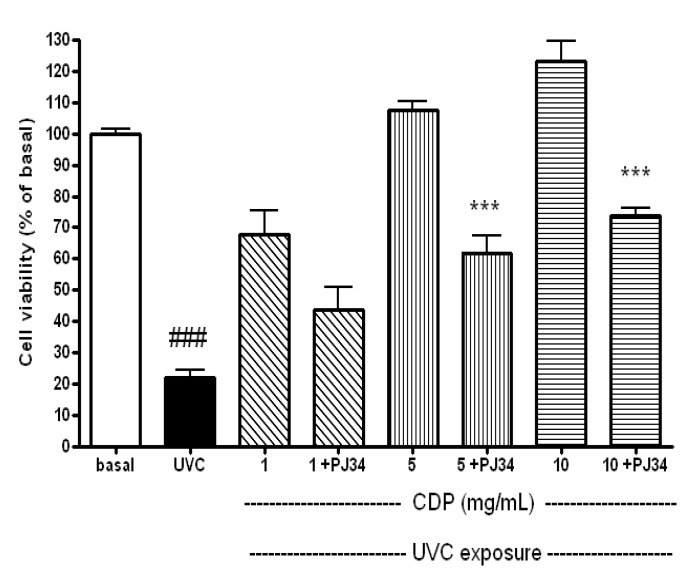
Counteractive effects of PJ34 on protective effects of CDP against to UVC-induced cytotoxicity. Skin fibroblasts were treated with CDP (1, 5 or 10 mg/mL) and PJ34 (20 μM) after UVC exposure with a total dose of 15 J/cm^2^ at 72 h prior to cell viability assay. Statistics are shown for UVC treated group ^###^ *p* < 0.005 compared to the basal and 5 +PJ34 and 10 +PJ34 ^***^ *p* < 0.005 compared to the CDP 5 mg/mL and 10 mg/mL-treated groups, respectively.

PARP-1 can be degraded by activated caspase-3, the cleaved PARP-1 is expected to lose its capability in protecting cells from UVC-induced damage and cause non-repaired DNA damage [13,17]. The expressions of cleaved PARP-1 (with 89 KDa) were clearly shown at 16 h after the UVC exposure (Figure 4). Again, the protein expression in the CDP-treated (5 and 10 mg/mL) cells were diminished which showed a protective effect on PARP-1. PJ34 (a specific inhibitor of PARP-1 activity) was added to CDP-treated fibroblasts to confirm that the protective effects of CDP against UVC-induced apoptosis were mediated through PARP-1 protection. The CDP protective effect was diminished and a decrease in cell viability was observed when the fibroblasts were treated with CDP and PJ34 (Figure 5, *p* < 0.005).

### 2.3. Inhibitory Effect of CDP on UVC-Induced DNA Damage

UV irradiation-induced DNA damage is usually recovered by nucleotide excision repair or base excision repair [29,30]. After UV exposure, cells activate p53 and stall the cell cycle for a purpose of repair [29]. If the damage is too severe, the cells will trigger apoptosis in order to remove these DNA damaged and potentially mutant cells.

**Figure 6:**
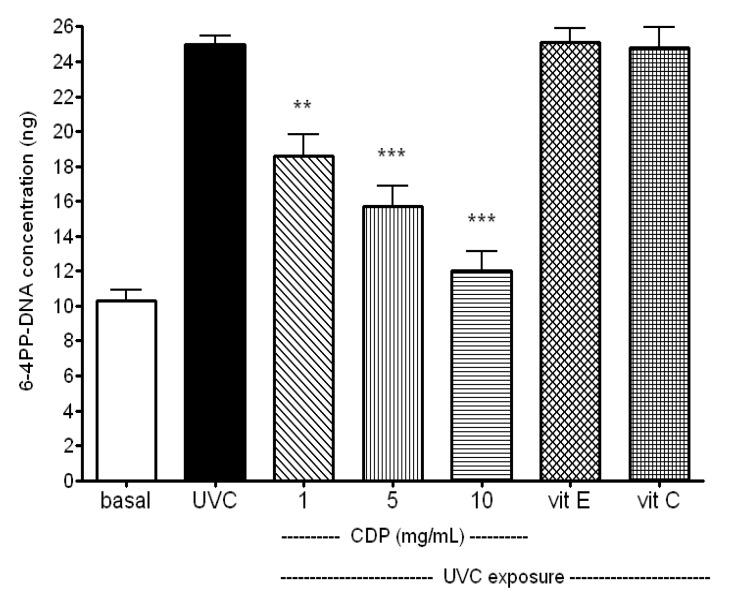
Inhibitory effects of CDP on UVC-induced DNA damage. Skin fibroblasts were treated with CDP (1, 5 or 10 mg/mL), vitamin C (150 μM), or vitamin E (25 μM) after UVC exposure with a total dose of 15 J/cm^2^ at 24 h prior to 6-4PP DNA concentration assay. Statistics are shown for 1 mg/mL CDP ^**^ *p* < 0.01; 5 and 10 mg/mL ^***^ *p* < 0.005 compared to the UVC treated group.

To elucidate whether UVC exposure induced DNA damage in the skin fibroblasts can be prevented by CDP, a pyrimidine (6-4) pyrimidone photoproducts (6-4PP) assay and a DNA laddering assay were performed. Results showed that the CDP-treated fibroblasts had much lower 6-4PP-DNA concentration in a dose-dependant manner (*p* < 0.01 for 1 mg/mL and *p* < 0.005 for 5 and 10 mg/mL of CDP, Figure 6) which means a lower extent of DNA damage upon UVC irradiation and CDP exerts a protective effect.

**Figure 7:**
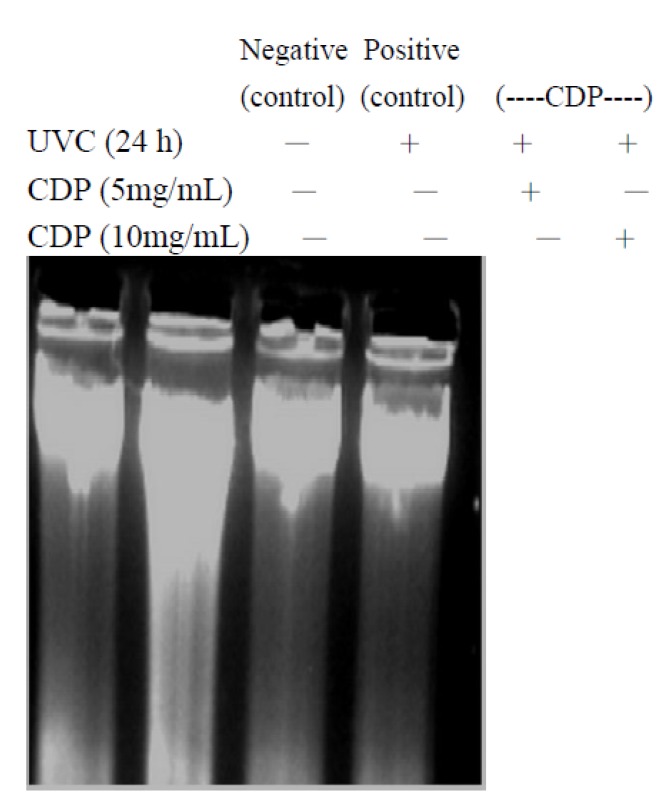
Inhibitory effects of CDP on UVC-induced DNA fragmentation**.** Skin fibroblasts were treated with CDP 5 and 10 mg/mL after UVC exposure with a total dose of 15 J/cm^2^ and cells were cultured at 24 h prior to harvesting for total DNA isolation.

In addition, CDP prevented apoptotic DNA cleavage after UVC exposure (Figure 7). Antioxidant vitamin E and C, on the other hand, showed not such protection UVC irradiation. The possible explanation is that UVC only induced delayed (more than 20 h) apoptotic mechanism which is initiated by DNA damage and it is not able to be prevented by vitamin E [31]. In addition other study showed that insulin-like growth factor also protected against UVC (20 J/cm^2^)-induced apoptosis through PKC pathway [32]. In our previous study also showed that CDP was able to inhibit PKC mediated MMP activity [25]. Protective effects of CDP against UVC could be mediated through DNA protection as shown in our results (Figure 5).

UVC irradiation causes DNA damage and triggers either cell cycle arrest or cell death to prevent proliferation of the damaged cells [29,30]. In this study, we demonstrate that CDP is able to protect cells against UVC-induced damage by preventing formation of phosphorylation of FADD and cleaved PARP-1 and inhibiting caspase-3 activity. *In vitro* study, *Chlorella* has also been shown to suppress caspase-3 activity [33]. Similarly, we observed caspase-3 activities were actually much lower in CDP-treated cells after the UVC exposure (Figure 2), which is consistent with the findings in preservation of cell viability against damage (Figure 1). UVC-induced apoptosis starts to activate CD95 receptor and recruit FADD [7]. The expression of phosphorylated FADD (the active form of FADD) was diminished in CDP-treated cells after UVC exposure indicates that the inhibition of UVC-induced apoptosis can be prevented by CDP and the pathway is via CD95 receptor route. Cell proliferation of CDP-treated groups (5 and 10 mg/mL) was much higher than that in a low dose of CDP (1 mg/mL) and even greater than the basal level. This can be due to the stimulating property of CDP on cell growth, since *Chlorella* has been shown to promote growth rate in animals [17] and spleen mononuclear cells proliferation [34].

After apoptotic signals activate caspase-3, it cleaves PARP-1 at Asp214 early in the apoptosis process. The cleaved PARP-1 loses its enzymatic activity, and the resulting N-terminal and C-terminal PARP-1 fragments accelerate apoptosis [35]. An inhibition of cleaved PARP-1 expression by CDP in the skin fibroblasts was found at 16 h after the UVC exposure which is in agreement with Lazebnik’s work that cleavage of PARP-1 started between 15 and 20 h after UVC irradiation [35]. The protection of PARP-1 from cleavage by CDP was further confirmed by adding PJ34 to CDP-treated fibroblasts after the UVC irradiation and observed a decrease in cell viability. Therefore, PARP-1 plays an important role in the protection of apoptosis by CDP before DNA damage is too severe beyond its ability to repair thus undergo apoptosis [29,30].

## 3. Experimental

### 3.1. Materials

Eagle-MEM culture medium was purchased from hyclone (Thermo Fisher Scientific Inc. Taipei, Taiwan). Monoclone antibodies of cleaved PARP-1 (^#^ 9546) and phosphorylated FADD (^#^ 2781) were obtained from Cell Signal Technology, Inc. (Taipei, Taiwan). DNA isolation kits were purchased from Qiagen (^#^ 13323, Taigen Bioscience Corporation, Taipei, Taiwan). UVC lamp (254 nm, Ultra-Violet products Model UVGL-58, Taipei, Taiwan) was purchased from local representatives. *Chlorella* dry powder was purchased from Gong-Bih Enterprise Co., LTD. (Taipei, Taiwan). Caspase-3 activity assay kits (K106-100) was obtained from BioVision (Taipei, Taiwan). Radioimmunoprecipitation assay buffer (R0278), vitamin C (A5960), vitamin E (T3634), PJ34 (P4365), and monoclonal anti-alpha-tubulin (T5168) were purchased from Sigma (Taipei, Taiwan). Superdex peptide HR 10/30 column (^#^ 285947) was purchased from Pharmacia (Taipei, Taiwan). Cell Proliferation Kit II (^#^ 1465015) was obtained from Roche Applied Science (Taipei, Taiwan). Polyvinylidene fluoride (PVDF) membrane was supplied by BioRad (Taipei, Taiwan). Horseradish peroxidase (RPN4301) and ECL detection system (RPN3001) were purchased from Amersham Pharmacia Biotech (Taipei, Taiwan). Total antioxidant capacity assay (STA-360) and DNA damage assay (STA-323) kits were obtained from Cell Biolabs (Taipei, Taiwan).

### 3.2. Cell Culture

Normal skin fibroblast 966SK (CRL 1881) cells were obtained from Bioresource Collection and Research Center (Taiwan) and cultured in Eagle-MEM supplemented with 1 mM sodium pyruvate and 10% FBS. The cells were maintained at 37 °C in a humidified atmosphere of 5% CO_2_–95% air. When cells reached above 80% confluence, subculture was conducted at a split ratio of 1:5.

### 3.3. Chlorella-Derived Peptide (CDP) Preparation

*Chlorella pyrenoidosa* dry powder was extracted with hot water (1: 10 ratio) for 30 min and then centrifuged at 9300 × g for 20 min. The resulting supernatant was firstly passed through 30 KDa ultra-membranes and then 5 KDa ultra-membranes in an Amicon stirred cell. The remnant was collected and freeze drying. A chromatogram of CDP on a Superdex peptide HR 10/30 column revealed a major molecular weight distribution of 430–1350 KDa [26].

### 3.4. Cell Viability Assay

Skin fibroblasts were seeded at a density of 1 × 10^4^ cells/well in a 96-well plate overnight. Subsequently, the culture media were replaced with PBS and the cells were exposed to UVC irradiation for 20 min at a dose rate of 12.5 mW/cm^2^ (with a total energy of 15 J/cm^2^). Energy of UVC exposure is comparable to others’ studies [7,28]. The fibroblasts were treated with of CDP (1, 5 and 10 mg/mL), vitamin C (150 μM), or vitamin E (25 μM dissolved in DMSO without exceeding 0.5% DMSO in working solutions) after the UVC exposure and incubated at 12, 24, 48, and 72 h at 37 °C. In some experiments, PARP-1 inhibitor, PJ34 (20 μM), was added to CDP-treated groups. Cell viability was assayed with a Cell Proliferation Kit II. Results were expressed as the relative cell viability (%) with respect to non-UVC exposed cells (referred to as basal). Concentrations of vitamin E and C were chosen based on previous finding [25].

### 3.5. Caspase-3 Activity Assay

Skin fibroblasts were treated with the same condition as described above. The cells were collected and lysed according to the indicated time points. The caspase-3 activity colorimetric assay is based on the hydrolysis of the peptide substrate acetyl-Asp-Glu-Val-Asp *p*-nitoaniline (Ac-DEVD-pNA) by caspase-3, resulting in the release of the p-nitroaniline (pNA) moiety. *p*-Nitroaniline has a high absorbance at 405 nm. The concentration of the pNA released from the substrate was calculated from the absorbance values at 405 nm.

### 3.6. Western Blotting Analysis of Phosphorylated FADD and Cleavage PARP-1

Skin fibroblasts were exposed to UVC as described above. After the UVC irradiation, the cells were harvested and lysed with radioimmunoprecipitation assay buffer 16 h later. Approximately 50 μg of cell lysate was boiled at 95 °C for 5 min in the sample buffer. The samples were then separated by 10% SDS-PAGE, followed by protein blotting onto PVDF membranes. The protein-blotted membranes were blocked with 5% (w/v) fat-free dry milk in PBS with 0.05% Tween 20 (PBS-T) overnight at 4 °C. They were then incubated with anti-phospho-FADD (Ser194), cleaved PARP-1 (Asp214) or housekeeping alpha-tubulin antibody at 1:1,000 dilution in PBS-T containing 1% bovine serum albumin overnight at 4 °C After washing three times for 5 min with PBS-T solution, blots were further incubated for 1 h at room temperature with goat anti-rabbit IgG antibody coupled to horseradish peroxidase at 1:2,000 dilution in 5% skim milk in PBS-T and washed three times in PBS-T before visualization. The expressions of the proteins were detected by an ECL detection system. Densitometric analysis was performed using the Alpha Imager 2000 Documentation & Analysis System (Alpha Innotech Corporation, San Leandro, CA, USA).

### 3.7. DNA Damage Assay

Absorption of UV light produces two predominant types of DNA damage, cyclobutane pyrimidine dimmers and pyrimidine (6-4) pyrimidone photoproducts (6-4PP). Skin fibroblasts were treated with the same condition as described above. The damaged DNA were measured as the quantitation of 6-4PP by using ELISA kits (Cell Biolabs STA-323).

### 3.8. DNA Fragment Assay

Cells (1 × 10^6^ cells) were exposed to UVC and harvested 24 h later. Whole DNA was then extracted with a DNA isolation kit and dissolved in TE buffer [10 μM Tris (pH 7.4), 10 μM EDTA (pH 8.0)] and subjected to 0.8% agarose gel electrophoresis at 50 V for 40 min and stained with ethidium bromide.

### 3.9. Statistics

Data from cytotoxicity and caspase-3 activity assays from each group (n ≥ 8) were combined from at least two different experimental days. A two-tailed Student’s unpaired t-test was used to compare the mean values of two populations of continuous data that were part of a normal distribution. Electrophoresis and Western blotting gel data from each group (n ≥ 3) were from at least three different experimental days, and a representative one is shown in results. 

## 4. Conclusions

CDP is a potent inhibitor against UVC-induced cytotoxicity. The mechanisms of prevention are through a significant reduction of UVC-induced cleaved PARP-1 formation and DNA damage. Combining our previous findings [25,26] that CDP has also proven to effectively decrease UVB-induced MMP-1 activity, CDP can be very useful in applications for dermal repair in UV-induced skin damage.
